# Circulating tumor DNA is a useful adjunct for Merkel cell carcinoma monitoring in the context of multiple metastatic malignancies

**DOI:** 10.1016/j.jdcr.2024.07.026

**Published:** 2024-08-10

**Authors:** Daniel Wenzel, Andrew M. Schuler, David C. Smith, Joseph R. Evans, Kelly L. Harms, Elisabeth A. Pedersen

**Affiliations:** a Department of Dermatology, Michigan Medicine and University of Michigan Medical School, Ann Arbor, Michigan; b Department of Pathology, Michigan Medicine and University of Michigan Medical School, Ann Arbor, Michigan; c Department of Medical Oncology, Michigan Medicine and University of Michigan Medical School, Ann Arbor, Michigan; d Department of Radiation Oncology, Michigan Medicine and University of Michigan Medical School, Ann Arbor, Michigan

**Keywords:** ctDNA, cutaneous oncology, dermatology, Merkel cell carcinoma, oncology

## Introduction

Merkel cell carcinoma (MCC) is an aggressive cutaneous cancer that carries a high risk of metastasis and recurrence.^1^ Advanced age and immune suppression significantly increase the risk of developing MCC and other aggressive malignancies.^2^ As high-risk patients including the elderly and transplant recipients are surviving longer with improved treatments, the incidence of multiple malignancies coexisting is anticipated to rise. Circulating tumor DNA (ctDNA) is emerging as a broadly useful test to monitor for treatment response and recurrence in several malignancies including MCC.^3^, ^4^, ^5^, ^6^ This case demonstrates the utilization of ctDNA in monitoring MCC burden in the scenario of multiple metastatic malignancies in a single patient.

## Case report

**Fig 1:**
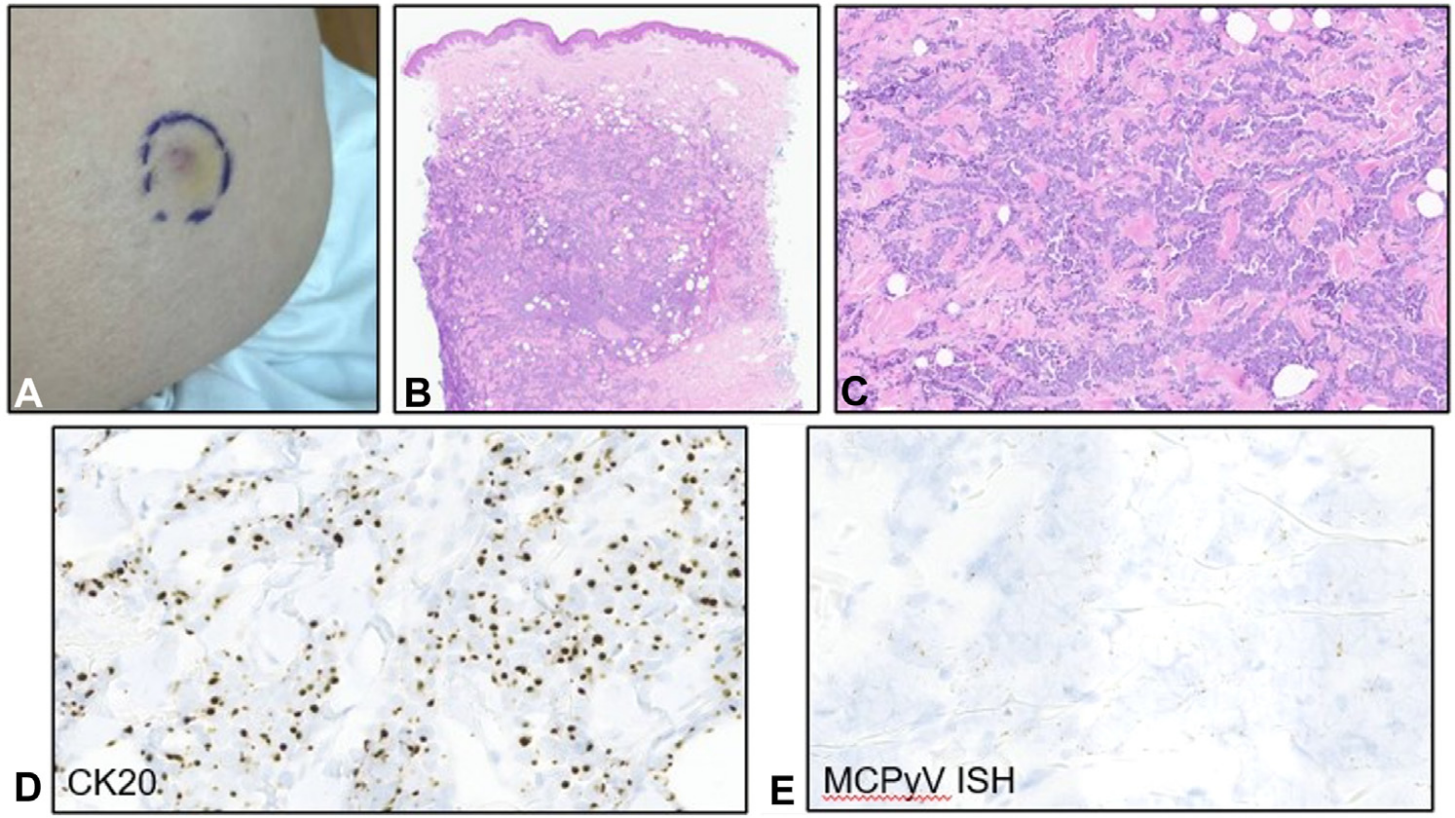
A patient with a known history of prostate cancer is diagnosed with Merkel cell carcinoma of the buttock. **A,** Clinical image of right buttock. **B** and **C,** Hematoxylin and eosin staining of the biopsy specimen at 20× and 200× magnification, respectively. **D,** Cytokeratin 20 (CK20) immunohistochemistry reveals positive staining in a dot-like pattern; 400× magnification. **E,** In situ hybridization for the Merkel cell polyoma virus (MCPyV) is positive; 400× magnification.

A 75-year-old man presented to dermatology for a concerning lesion. He had a history of prostate cancer metastatic to bone diagnosed over 5 years prior, treated with androgen deprivation therapy, corticosteroids, and radiation. Examination showed a 2 cm nodule on his right buttock (Fig 1, *A*). Biopsy revealed a tumor comprised of cords and nests of small, round blue cells infiltrating deeply within the reticular dermis (Fig 1, *B*). Cells with a high nuclear to cytoplasmic ratio, characteristic neuroendocrine chromatin pattern, and scattered mitoses were present (Fig 1, *C*). Cytokeratin 20 immunohistochemical stain showed a dot-like pattern at the nuclear periphery (Fig 1, *D*), and in-situ hybridization for Merkel cell polyoma virus was positive (Fig 1, *E*), consistent with MCC. He underwent wide local excision and sentinel lymph node biopsy which revealed clear margins at the primary site and occult metastatic disease in a right inguinal lymph node. Imaging did not reveal additional sites of disease, consistent with stage IIIA (pT2 pN1a(sn) M0).

**Fig 2:**
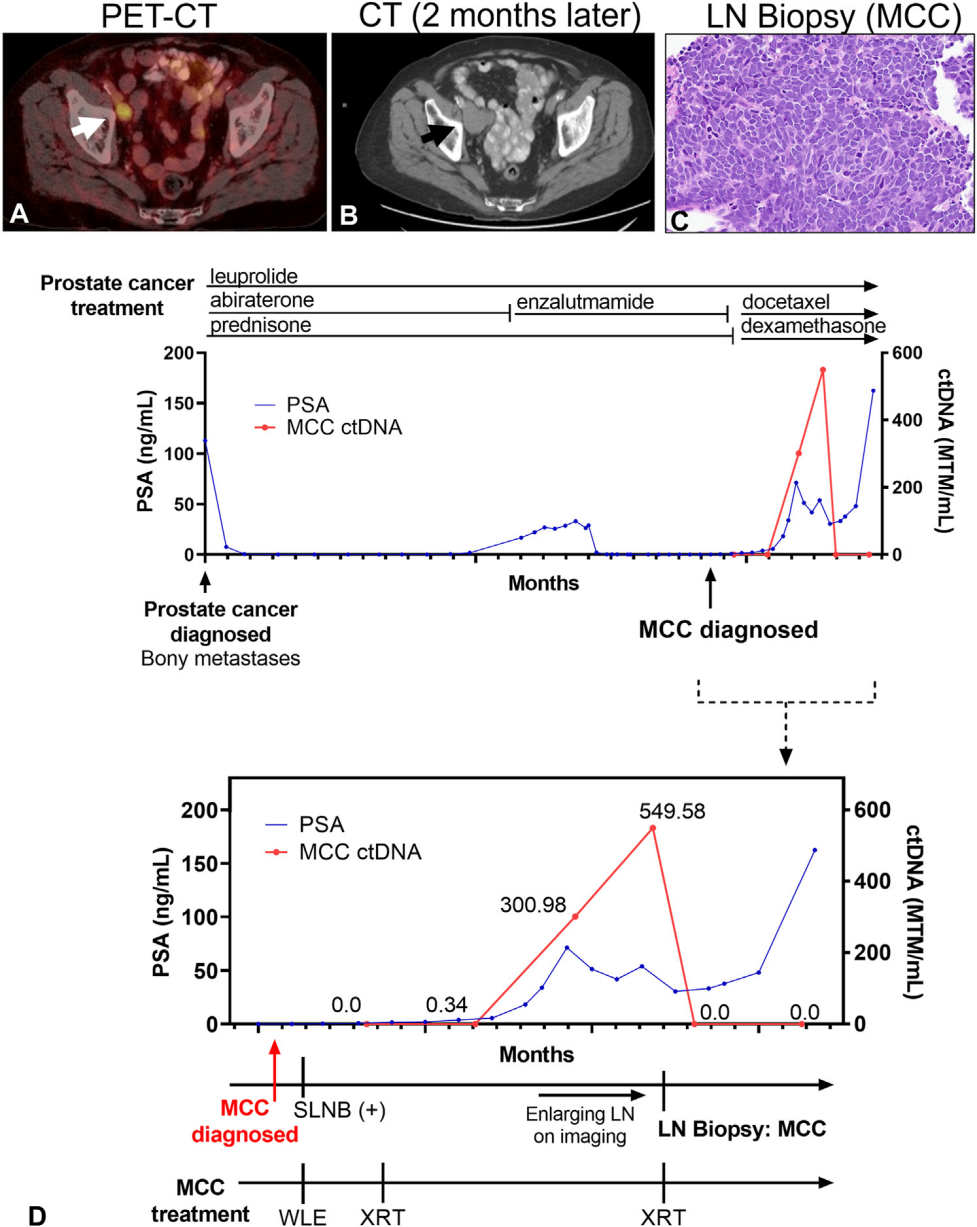
Circulating tumor DNA correlates with Merkel cell carcinoma (MCC) tumor burden. **A**, 68-Gallium prostate specific membrane antigen-targeted positon emission tomography (^6^^8^Ga-PSMA-PET) reveals a new avid inguinal lymph node approximately 6 months following MCC treatment, which continued to enlarge on computed tomography (CT) imaging (**B**) over the course of 2 months. Biopsy of the enlarged lymph node showed MCC recurrence diagnosed by (**C**) hematoxylin and eosin staining of the lymph node; 200×. **D,** A summary of prostate cancer treatment (*top panel*) and MCC treatment (*bottom panel*) reveals correlation between MCC circulating tumor DNA (ctDNA) values and tumor burden.

Monitoring with MCC-specific ctDNA was initiated 4 weeks after wide local excision and was undetectable with a value of zero mean tumor molecules per milliliter (0 MTM/mL). Three weeks later, the primary tumor site and right inguinal lymph node basin were treated with adjuvant radiation. Nine weeks after local radiation, ctDNA increased to 0.34 MTM/mL. Total body skin and lymph node examination were normal. Ultrasound of the pelvis revealed no abnormalities. He simultaneously developed PSA elevation. Gallium 68 prostate specific membrane antigen-targeted positon emission tomography (^6^^8^Ga-PSMA-PET) revealed progression of known bony prostate carcinoma lesions and new uptake in a 1.5 cm right external iliac lymph node (Fig 2, *A*). Docetaxel for recurrent prostate cancer was initiated. After 2 months, PSA and bony metastases subsequently decreased, however MCC ctDNA MTM/mL increased to 300.96 MTM/mL. Imaging revealed an increase in size of the previously noted external iliac lymph node (Fig 2, *B*). Biopsy revealed recurrent MCC (Fig 2, *C*). The recurrence was treated with radiotherapy and MCC ctDNA became undetectable (0 MTM/mL) 3 weeks after initiation of radiation, despite ongoing prostate cancer progression. Follow-up imaging confirmed resolution of lymphadenopathy. A summary of his diagnostic testing and treatment course is shown in Fig 2, *D*.

## Discussion

MCC is an aggressive, difficult to treat malignancy with high rates of recurrence and poor survivability in patients with metastatic disease. MCC patients are also at risk for multiple malignancies, and MCC displays an increased risk of arising as a secondary cancer.^2^^,^^7^ This can create challenges for surveillance and monitoring treatment response, particularly in cases with multiple cancers. Sequential analysis with ctDNA is a promising approach.^4^, ^5^, ^6^ Whole-exome sequencing from formalin-fixed tumor specimens is used to create individualized serum tests targeted towards tumor-specific DNA. In a study of 125 patients, all patients with clinically evident MCC had positive ctDNA, demonstrating up to 100% sensitivity.^5^ Prior to ctDNA, our ability to detect tumor recurrence and treatment response using serum or whole blood-based tests has been limited to detecting circulating antibodies in patients with Merkel cell polyoma virus positive tumors, which is not sensitive and not applicable in patients with virus-negative tumors. In cases with multiple malignancies, traditional radiologic imaging does not differentiate well, and in the patient described, elevated ctDNA correlated with MCC tumor burden. Elevated ctDNA was observed prior to MCC detection on imaging, allowing for early detection of subclinical recurrence despite high-sensitivity imaging suggesting prostate cancer progression in the lymph node. There are important limitations to ctDNA to consider: a biopsy is still required to confirm diagnosis and false negative tests may lead to false reassurance and inadequate treatment. In addition, different ctDNA tests might perform differently among specific malignancies and manufacturers. Our patient had ctDNA testing through Signatera, which is 1 of at least twelve known biotechnology companies offering commercially available ctDNA testing at this time. Routine ctDNA testing is not typically covered by most insurances and may not yet be easily accessible to all patients; however, this may evolve as more data emerges. Future studies investigating MCC ctDNA sensitivity and specificity are still needed to provide information for the applications of ctDNA in MCC, which will help determine best practices for ctDNA monitoring, including need for baseline testing and thresholds for clinical intervention. Currently, preliminary results are promising in a growing number of clinical scenarios.

## Conflicts of interest

None disclosed.
